# Supplementary material for: How do various leadership practices affect professional learning communities? The mediating role of principals’ perceived trust by teachers
Source: PLoS One. 2025 May 6;20(5):e0323055. doi: 10.1371/journal.pone.0323055 (PMC12054885; doi:10.1371/journal.pone.0323055)
Supplement: Attachment — Submitted filename: Response to Reviewers.doc [file pone.0323055.s002.doc]

**Response to Reviewers**

**Dear Editors,**

We wish to thank the anonymous reviewers for their careful reading of the manuscript and helpful comments. We believe we have used these comments in straightforward way to improve the research. Our detailed responses to the review comments are presented in a point by point format. Related changes are highlighted in red font in the main text.

**Reviewer 1 :**

Comments to the Author

The manuscript is methodologically rigorous, addresses a relevant topic, and provides original contributions. While focused on the Chinese context, it meets the standards for publication. Emphasising global implications could further strengthen the discussion.

Response: Thank you for the positive comments. We are pleased that our manuscript has been recognized for its methodological rigor and original contributions. We have taken your suggestion to heart and have revised the manuscript to better emphasize its global implications. Specifically, we have made the following changes in the “**Introduction**” “**Conclusion and Discussion**” and “**Implications**” sections, and have also added relevant international literature to support our views and findings. These revisions are aimed at enhancing the international impact of our study. (please see page 3/5/20/23).

***Introduction Section:***

We have added the following sentences to highlight the global relevance of our study:

"Understanding how PLCs are fostered and developed in diverse settings, including China, can offer valuable insights for global educational reform efforts." (please see page 3).

"By exploring these questions, the study not only enhances our understanding of PLCs within the Chinese educational context but also contributes to the broader global discourse on the role of principal leadership and trust in promoting the development of PLCs." (please see page 5).

These additions emphasize that our research on PLCs in China can provide broader insights applicable to educational reforms worldwide.

***Conclusion and Discussion Section*:**

We have expanded the discussion on the theoretical contributions of our study:

"The findings enrich the international literature on the relationship between principal leadership and PLCs by offering valuable insights from the Chinese experience, contribute to the application of social exchange theory in explaining how leadership practices affect PLCs and introduce the noteworthy perspective of principals to research in this field. Importantly, these insights, grounded in the Chinese context, offer a potential foundation for understanding similar dynamics between leadership, trust, and PLCs development in diverse global educational settings." (please see page 20).

This integration of Chinese educational practices with global research underscores the broader applicability of our findings.

***Implications Section:***

We have emphasized the broader applications of our study:

"The present study has important implications for school leaders and policy makers, especially those in China, and suggests broader applications for educational settings globally." (please see page 23).

By highlighting the broader applications, we aim to show that our research can inform educational practices beyond China.

**Reviewer 2 :**

Comments to the Author

Overall, I find this study to be well-designed and and clearly presented. The research addressed important gaps in the literature and provided valuable insights into the mechanism through which leadership practices influence professional learning communities. This study is well-designed and clearly presented, addressing important gaps in the literature on how leadership practices influence PLCs through the mediating role of teachers' trust in principals. The use of social exchange theory and robust quantitative methods, including hierarchical regression and validation techniques, ensures the reliability and validity of the findings. The manuscript is logically structured, with clear articulation of findings and practical implications. Overall, this research makes a valuable contribution to the field.

Response: Thank you for the positive comments.

Minor Suggestions: Some references were relatively old. I suggest the authors to add more recent studies, particularly those related to the relationships between leadership practices and PLCs, to enhance the timeliness of the research.

Response: Thank you for your suggestion. We have carefully reviewed our references and added several studies published in the past five years to enhance the timeliness and relevance of our research. The corresponding citations in the main text have been updated and highlighted in red to clearly indicate these changes.

Minor Suggestions: The description of control variables was brief. I suggest the authors provide more detailed information about these variables. For example, what specific positions were included under "position"? What levels of schools were categorized under "school level"? Clarifying these details will help readers better understand the scope and precision of the study's control mechanisms.

Response: Thank you for your suggestion. We have expanded the description of the control variables in the "**Method**" section to provide more clarity. The revised text is as follows:

“Principals' gender (1 = male, 2 = female), age (1 = ≥51; 2 = 41–50; 3 = ≤40), position (1 = elementary schools; 2 = middle schools; 3 = high schools; 4 = other schools), school level (1 = elementary schools; 2 = middle schools; 3 = high schools; 4 = other schools), geographic location (1 = urban areas; 2 = suburban and rural areas), and school type (1 = model school; 2 = ordinary school) were included in the questionnaire as control variables. These control variables were transformed into dummy variables and then added to the data analysis.” (please see page 13).

Please include a copy of Table 3 which you refer to in your text on page 18.

Response: Thank you for your attention to our manuscript. We have reviewed the content and found that the table caption "*Mediation analysis of principals' perceived trust by teachers on the relationships between leadership practice components and PLCs*" was incorrectly labeled as Table 2. Following your guidance, we have now corrected this to Table 3.
